# Serum Maresin-1 and Resolvin-D1 Levels as Non-Invasive Biomarkers for Monitoring Disease Activity in Ulcerative Colitis

**DOI:** 10.3390/diagnostics15070834

**Published:** 2025-03-25

**Authors:** Selim Demirci, Semih Sezer

**Affiliations:** Department of Gastroenterology, Dr. Abdurrahman Yurtaslan Oncology Training and Research Hospital, 06200 Ankara, Türkiye; ssezer1970@hotmail.com

**Keywords:** disease activity, ulcerative colitis, biomarkers, inflammation, maresins, resolvins, specialized pro-resolving mediators

## Abstract

**Background:** Specialized pro-resolving lipid mediators (SPMs), such as maresins and resolvins, play a key role in resolving inflammation and repairing tissues. This study aimed to evaluate whether maresin-1 (MaR1) and resolvin-D1 (RvD1) could serve as serum non-invasive biomarkers for monitoring disease activity in ulcerative colitis (UC). **Methods:** This cross-sectional study included 60 UC patients (30 active, 30 remission) and 30 healthy controls. Disease activity was assessed using the Mayo Endoscopic Subscore (MES). Inflammatory indices, including the neutrophil–lymphocyte ratio (NLR), monocyte–HDL cholesterol ratio (MHR), platelet–lymphocyte ratio (PLR), CRP–lymphocyte ratio (CLR), CRP–albumin ratio (CAR), systemic inflammation response index (SIRI), and systemic immune-inflammation index (SII), were calculated. Plasma MaR1 and RvD1 levels were measured via enzyme-linked immunosorbent assay (ELISA). Receiver operating characteristic (ROC) analysis was performed to evaluate biomarker accuracy. **Results:** CRP, NLR, PLR, CLR, CAR, SIRI, and SII were significantly elevated in active UC, whereas MaR1 and RvD1 were lower compared to remission and controls (*p* < 0.05). MaR1 levels were lower in the remission group than in controls. ROC analysis demonstrated high area under the curve (AUC) values for RvD1 (0.906), CAR (0.872), CLR (0.861), and CRP (0.858) in distinguishing active UC from remission, and for CRP (0.944), CAR (0.939), CLR (0.939), RvD1 (0.928), and MaR1 (0.889) in distinguishing active UC from controls. The specificity for detecting active UC was 60% for MaR1 and 80% for RvD1. Both RvD1 and MaR1 showed a negative correlation with the MES, with RvD1 demonstrating a stronger correlation (r = −0.754, *p* < 0.001). **Conclusions:** RvD1 shows a strong negative correlation with disease severity in ulcerative colitis, while low MaR1 levels in remission may indicate subclinical inflammation. Although MaR1 and RvD1 are not disease-specific, their role in inflammation resolution suggests they may complement conventional inflammatory markers for more comprehensive UC monitoring.

## 1. Introduction

Ulcerative colitis (UC), a persistent inflammatory condition of the bowel, has been experiencing a rising prevalence globally [1]. Its hallmark symptoms include bloody stools, diarrhea, weight loss, and abdominal pain. Although the exact etiology of UC remains unclear, excessive activation of the immune system and overproduction of pro-inflammatory cytokines contribute to mucosal damage and persistent inflammation [2]. Due to its relapsing and remitting nature, achieving clearly defined therapeutic goals is crucial for effective disease management [3]. Early detection of disease activity is essential, as it significantly reduces the need for surgical intervention and lowers mortality rates in patients with severe UC [4]. Endoscopy remains the gold standard for diagnosing UC and assessing disease activity. However, the invasive nature of endoscopic and histological procedures, coupled with low patient acceptance, render them impractical and less desirable for routine monitoring [5]. As a result, there is a growing need for reliable serum non-invasive biomarkers that can aid in disease monitoring, guide therapeutic decisions, and minimize the dependence on repeated endoscopic procedures.

In clinical practice, serum non-invasive markers such as erythrocyte sedimentation rate (ESR) and C-reactive protein (CRP) are widely used for diagnosing and monitoring disease activity in UC. Although ESR and CRP levels may change during active UC, they are nonspecific indicators that can also be affected by various other inflammatory conditions. Furthermore, since UC-associated inflammation is primarily localized to the intestinal mucosa and shows only a weak correlation with systemic inflammation, these markers have limited utility in accurately reflecting endoscopic disease activity [6]. As is well established, inflammation is a major determinant in the pathogenesis and the progression of UC, leading to significant alterations in peripheral blood leukocyte levels. Therefore, several inflammatory markers associated with complete blood count (CBC) and certain biochemical parameters, such as the neutrophil–lymphocyte ratio (NLR), monocyte–HDL cholesterol ratio (MHR), platelet–lymphocyte ratio (PLR), CRP–lymphocyte ratio (CLR), CRP–albumin ratio (CAR), systemic inflammation response index (SIRI), and systemic immune-inflammation index (SII), have been observed in some studies as indicators of clinical disease activity in UC [7,8,9,10]. Despite their potential utility, no single biomarker has been identified as an optimal indicator of mucosal inflammation in UC, as currently available markers lack specificity [11].

Specialized pro-resolving lipid mediators (SPMs), including maresins, resolvins, and protectins, are a group of highly bioactive lipids known for their anti-inflammatory activities [12]. SPMs are synthesized within human body fluids and organs, contributing to the resolution of inflammation, promotion of wound healing, and neuroprotection by exerting their effects on macrophages, neutrophils, lymphocytes, epithelial cells, and endothelial cells. SPMs exert their effects even at picogram-to-nanogram concentrations by selectively binding to stereospecific receptors on immune cells and activating organ-specific protective pathways [13]. Maresin-1 (MaR1) is a macrophage-derived lipid mediator synthesized from docosahexaenoic acid (DHA) and recognized for its critical role in inflammation resolution. Multiple studies have revealed that MaR1 demonstrates anti-inflammatory properties by limiting neutrophil migration and cytokine secretion by stimulating Th1 cells, Th17 cells, and CD8+ T cells [14]. Furthermore, it has been shown to suppress the transcription factors Rorc and T-bet, and inhibit the differentiation of Th1 and Th17 cells, while concurrently fostering the growth of Foxp3+ regulatory T (Treg) cells through the GPR32 receptor pathway [15]. Resolvin-D1 (RvD1) is a recently identified DHA-derived lipid molecule that exerts a crucial role in resolving inflammation by regulating neutrophil activity, clearing apoptotic polymorphonuclear neutrophils (PMNs), and reducing the release of chemokines and pro-inflammatory cytokines [16]. Blood levels of MaR1 and RvD1 have been studied in various acute and chronic inflammatory diseases, including Hashimoto’s thyroiditis (HT) [17], acute pancreatitis [18], rheumatoid arthritis (RA) [19], nonalcoholic fatty liver disease (NAFLD) [20], and irritable bowel syndrome (IBS) [21], consistently demonstrating significantly lower levels compared to healthy controls. Unlike traditional biomarkers that merely indicate ongoing inflammation, MaR1 and RvD1 actively promote their resolution, offering distinct advantages in inflammatory disease monitoring. UC is a chronic inflammatory disorder characterized by an exaggerated immune response against the colonic mucosa, driven by an imbalance between pro-inflammatory cytokines, excessive immune cell infiltration, and impaired resolution of inflammation [22]. Traditionally, UC pathogenesis has been linked to excessive activation of innate and adaptive immune pathways, particularly involving cytokines such as tumor necrosis factor-alpha (TNF-α), interleukin-6 (IL-6), and interleukin-1β (IL-1β) [23]. However, growing evidence highlights the importance of endogenous regulatory systems that counterbalance inflammation, including the melanocortin system (MCS). The MCS, through melanocortin receptors (MC1R–MC5R), modulates cytokine secretion, immune cell migration, and intestinal barrier integrity, contributing to the regulation of mucosal inflammation in UC [24]. Studies have shown that melanocortin receptor activation reduces pro-inflammatory cytokines such as TNF-α, IL-6, and IL-1β, which are implicated in UC pathogenesis [25]. The capacity of these receptors to respond to endogenous agonists such as [Nle^4^, DPhe^7^]-α-Melanocyte-Stimulating-Hormone (MSH), α-MSH, and γ-MSH highlights their crucial role in modulating inflammatory responses [26]. Notably, Montero-Melendez emphasized α-MSH as an example of an ‘endogenous-based pro-resolving therapy’, underscoring its ability to actively terminate inflammation rather than merely suppress it [27]. In this context, a striking parallel can be drawn between the melanocortin system and SPMs, including MaR1 and RvD1. Based on this, we hypothesize that MaR1 and RvD1, as key mediators of inflammation resolution, may provide novel insights into UC activity by reflecting the balance between pro-inflammatory and pro-resolving mechanisms.

This cross-sectional study aims to evaluate the plasma concentrations of MaR1 and RvD1 in patients with UC and healthy controls, exploring their potential as biomarkers for disease monitoring and their association with disease activity.

## 2. Materials and Methods

### 2.1. Study Population

This study was conducted at the Gastroenterology outpatient clinic of Abdurrahman Yurtaslan Oncology Training and Research Hospital between April and June 2024. Patients who consecutively presented to the clinic during this period and were deemed eligible based on the inclusion criteria were invited to participate after providing written informed consent. Endoscopic evaluations were performed as part of routine clinical assessment, and blood samples from eligible patients for MaR1 and RvD1 measurements were collected on the same day to ensure a close temporal correlation between biomarker levels and endoscopic disease activity.

Disease activity was classified using the Mayo Endoscopic Score (MES), which evaluates endoscopic disease activity on a scale from 0 to 3. An MES of 0 indicates normal or healed mucosa, MES 1 represents erythema and mild friability without spontaneous bleeding; MES 2 is characterized by marked erythema, erosions, and friability, and MES 3 denotes severe inflammation with spontaneous bleeding and deep ulcerations [28]. Patients with MES 2 and 3 were considered the active UC group, while those with MES 0–1 were classified as the remission UC group. To maintain group homogeneity and ensure comparability, the active group included patients currently presenting with extensive colitis, while the remission group comprised patients with a documented history of extensive colitis at initial diagnosis. The control group consisted of healthy individuals who visited our outpatient clinic for health check-ups during the same period. Demographic and clinical data for patients, such as sex, age, body mass index (BMI), disease course, endoscopy results, disease extent, and detailed medication history, were recorded.

Patients under the age of 18 or over 80, as well as those with malignancy, chronic kidney disease, coronary heart disease, diabetes mellitus, rheumatologic diseases, neurodegenerative diseases, collagen tissue disorders, a history of colectomy, acute or chronic infections, those who were pregnant or lactating, or those unwilling to participate in the study, were excluded. The control group consisted of volunteers aged 18 to 80 with no known comorbidities, malignancies, infections, or pregnancy/lactation status. Participants who had used corticosteroids, biologic medications, omega-3 supplements, acetylsalicylic acid, or nonsteroidal anti-inflammatory drugs (NSAIDs) within the past week were also excluded. To minimize potential dietary influences on MaR1 and RvD1 levels, participants adhering to diets rich in omega-3 (e.g., Mediterranean diet and marine-based diets) or high in polyphenols (e.g., plant-based diets) were excluded from the study. Conversely, individuals following omega-6-rich Western diets, which may suppress SPM production by promoting pro-inflammatory eicosanoids, were also excluded to ensure consistency in biomarker assessment [12,29,30].

The Hospital Ethics Committee gave its clearance for the study to be carried out (Approval No. 2024-01/07). All participants received full details about the study, and their written consent was acquired. The ClinicalTrials registration number is NCT06652464.

### 2.2. Data Acquisition and Laboratory Analysis

Peripheral venous blood samples were collected from enrolled patients following a minimum of eight hours of fasting. The following laboratory parameters were measured: CRP (mg/L; normal range: <5 mg/L), ESR (mm/h; normal range: 0–20 mm/h), neutrophil count (×10^3^/μL; normal range: 1.8–7.7 × 10^3^/μL), lymphocyte count (×10^3^/μL; normal range: 1.1–3.2 × 10^3^/μL), monocyte count (×10^3^/μL; normal range: 0.2–0.9 × 10^3^/μL), platelet count (×10^3^/mm^3^; normal range: 150–450 × 10^3^/mm^3^), albumin (g/dL; normal range: 3.5–5.0 g/dL), HDL cholesterol (mg/dL; normal range: >40 mg/dL), and inflammatory indices were calculated accordingly. An extra 2 mL blood sample was obtained specifically for MaR1 and RvD1 measurements and transferred into 5 mL EDTA tubes containing gel. The samples were centrifuged under sterile conditions at 1000 rpm for 20 min to separate the serum. The serum samples were kept in a deep freezer at −80 °C in dry, clean Eppendorf tubes until analysis. All samples were processed and analyzed in a single batch to ensure consistency. Plasma MaR1 and RvD1 levels were measured using enzyme-linked immunosorbent assay (ELISA) kits, with detection ranges of 7.81–500 pg/mL for MaR1 and 15.63–1000 pg/mL for RvD1, respectively. The MES was determined through standardized colonoscopy procedures performed by a single experienced gastroenterologist and documented in hospital records.

### 2.3. Inflammatory Indices

The inflammatory indices were determined as follows: NLR = neutrophils (×10^3^/μL)/lymphocytecount (×10^3^/μL); PLR = platelet (×10^3^/mm^3^)/lymphocyte count (×10^3^/μL); MHR = monocytes (×10^3^/μL)/HDL cholesterol (mg/dL); CLR = CRP (mg/L)/lymphocytes (×10^3^/μL); CAR = CRP (mg/L)/albumin (g/dL); SII = neutrophils (×10^3^/μL) multipliedby platelets (×10^3^/mm^3^), divided by lymphocytes (×10^3^/μL); and SIRI = neutrophils (×10^3^/μL) multiplied by monocytes (×10^3^/μL), divided by lymphocytes (×10^3^/μL).

### 2.4. Statistical Analyses

A priori power analysis was conducted using GPower 3.1 (Kiel, Germany) to estimate the required sample size. Based on previous studies [7] examining inflammatory markers in UC, an effect size of 0.40, a power of 80% (β = 0.20), and a significance level of 0.05 were applied. The analysis indicated that a minimum of 84 participants (28 per group) was needed to detect statistically significant differences between groups. Thus, our final sample size of 90 participants (30 per group) was considered sufficient for statistical comparisons. The SPSS software, version 27 (IBM Corp., Armonk, NY, USA) and R version 3.6.1 (R Foundation for Statistical Computing, Vienna, Austria), were used for all statistical analyses. The Shapiro–Wilk test was applied to assess the normality of the data distribution. Continuous data were given as mean ± standard deviation or median (IQR)/median (Q1–Q3) depending on their distribution, whereas categorical variables were reported as counts and percentages. The chi-square test was employed to analyze and compare categorical variables. Group comparisons for normally distributed data were conducted using one-way ANOVA with subsequent LSD posthoc analysis, whereas the Kruskal–Wallis test was utilized for data that did not meet normality assumptions. For subgroup analyses comparing 5-ASA users and non-users, the Mann–Whitney U test was used due to the non-normal distribution of the data and the small sample size in the subgroups. To assess the ability of each biomarker to differentiate the active UC group from the remission and control groups, receiver operating characteristic (ROC) curve analysis was performed. To enhance generalizability and mitigate potential optimism bias, area under the curve (AUC) values were derived through K-Fold Cross-Validation (K = 5) and reported across test sets. The DeLong method was applied to compare biomarkers with AUC values above 0.800, as higher AUC thresholds indicate stronger diagnostic performance. Sensitivity, specificity, and cutoff values were derived from the original dataset. By determining the highest Youden index, the ideal cutoff value for each ratio was identified. The correlations between serum MaR1, RvD1, other inflammatory indices, and the MES were determined using either Spearman’s tests based on the distribution characteristics of the parameters. To account for multiple hypothesis testing in the ROC and correlation analyses, we applied a false discovery rate (FDR) correction via the Benjamani–Hochberg (BH) method to control for potential Type I errors. A threshold of *p* < 0.05 was used to determine statistical significance.

## 3. Results

**Figure 1:**
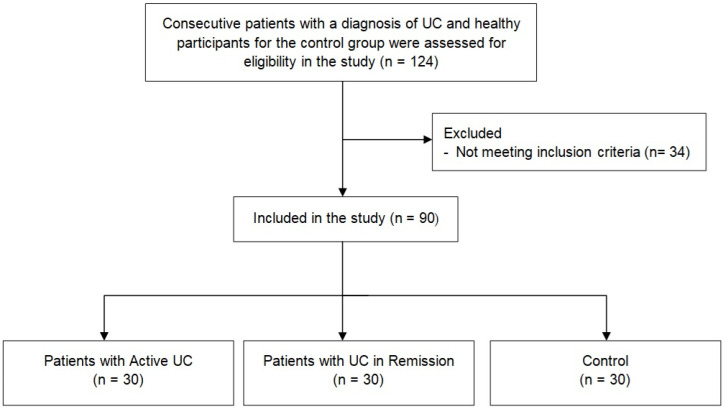
Flowchart of the study. UC; ulcerative colitis.

The study enrolled 60 individuals diagnosed with UC, comprising 30 in the active phase and 30 in remission, along with 30 healthy participants as controls (Figure 1). Table 1 displays an overview of the patients’ clinical and demographic characteristics. Age, sex, BMI, and smoking habits did not vary significantly between the disease groups (*p* > 0.05). While there was a significant difference in PLR values between the active UC and control groups, CRP, NLR, CLR, CAR, SII, SIRI, and RvD1 levels showed significant differences between the active UC group and both the remission and control groups. MaR1 levels were the only parameter to show a statistically significant decrease across all groups.

A sub-analysis was performed to evaluate whether mesalazine (5-ASA) therapy influences MaR1 and RvD1 levels in both the active and remission UC groups, as shown in Table 2. In the active UC group, MaR1 levels were 114.37 pg/mL (97.22–132.44) in 5-ASA users (*n* = 18) and 101.08 pg/mL (91.14–113.99) in non-users (*n* = 12) (*p* = 0.146). Similarly, RvD1 levels were 294.19 pg/mL (253.25–305.91) in 5-ASA users and 283.33 pg/mL (248.29–294.59) in non-users (*p* = 0.267). In the remission group, MaR1 levels were 123.93 pg/mL (118.93–132.08) in 5-ASA users (*n* = 24) and 123.62 pg/mL (117.21–132.38) in non-users (*n* = 6) (*p* = 0.820), while RvD1 levels were 359.14 pg/mL (308.59–391.04) and 332.62 pg/mL (262.93–381.11), respectively (*p* = 0.273). These results suggest that 5-ASA therapy does not significantly influence MaR1 or RvD1 levels in either active or remission phase UC patients.

**Figure 2:**
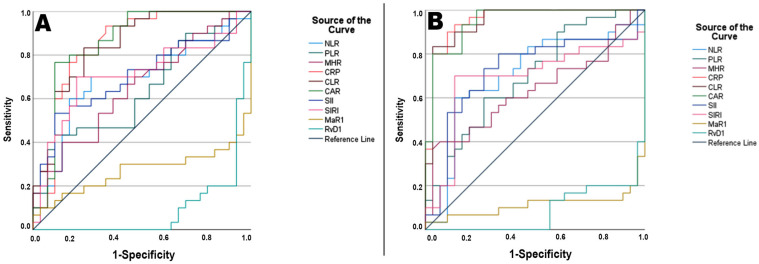
(**A**) ROC curve for distinguishing active patients from those in remission. (**B**) ROC curve for distinguishing active patients from controls. NLR, neutrophil-to-lymphocyte ratio; PLR, platelet-to-lymphocyte ratio; MHR, monocyte-to-high-density lipoprotein cholesterol ratio; CRP, C-reactive protein; CLR, C-reactive protein-to-lymphocyte ratio; CAR, C-reactive protein-to-albumin ratio; SII, systemic immune-inflammation index; SIRI, systemic inflammation response index; MaR1, maresin-1; and RvD1, resolvin-D1. ROC curves were plotted using AUC values derived from the original dataset.

The ability of inflammatory markers to differentiate active UC from remission and controls was assessed using ROC curve analysis. The results are presented in Table 3 and Table 4. In the active–remission group, the ROC analysis showed that an RvD1 value of <303.23 pg/mL had an AUC of 0.906 (sensitivity 93.30%, specificity 80.00%), higher than other inflammatory indices. Subsequently, CAR (AUC: 0.872, sensitivity 76.70%, specificity 90.00%) and related inflammatory parameters [CLR (AUC: 0.861), CRP (AUC: 0.858)] were found to have high sensitivity and specificity in differentiating active cases from remission. In the active–control group, CRP (AUC: 0.944, sensitivity 93.30%, specificity 86.70%) was the most effective, followed by the CRP-related inflammatory parameters CLR (AUC 0.939, sensitivity 83.30%, specificity 96.70%and CAR (AUC 0.939, sensitivity 93.30%, specificity 83.30%), which had higher sensitivity and specificity compared to other parameters in distinguishing active cases from controls. To differentiate active UC patients from healthy controls, the optimal cutoff values based on the highest Youden index were determined as <129.88 for MaR1 (sensitivity: 96.70%, specificity: 80.00%) and <303.35 for RvD1 (sensitivity: 96.70%, specificity: 80.00%) (Figure 2). Despite observed differences in AUC values between groups, DeLong analysis revealed no statistically significant discriminative differences among parameters with an AUC above 0.800 (*p* > 0.05).

Table 5 presents the correlation analysis between inflammatory parameters and the MES. Spearman’s correlation analysis revealed statistically significant negative correlations between MaR1 (r = −0.407, *p* = 0.002) and RvD1 (r = −0.754, *p* < 0.001) with MES in UC patients. RvD1, CAR, and CLR exhibited a strong correlation with MES (r > 0.6); among all parameters, RvD1 demonstrated the strongest negative correlation with MES.

## 4. Discussion

Our study is the first to investigate MaR1 and RvD1 levels in patients diagnosed with UC, comparing them with other inflammatory indices. We observed significant increases in CRP, NLR, CLR, CAR, SII, and SIRI levels in the active UC group compared to the remission and control groups, while RvD1 and MaR1 levels were significantly reduced. These findings align with previous research on systemic inflammation in UC and reinforce the robustness of our study by confirming the characteristic pattern of inflammatory marker elevation in active disease and reduction in remission and controls. While RvD1, MaR1, CRP, CAR, and CLR demonstrated potential for differentiating between groups, none showed a distinct diagnostic advantage over the others. RvD1, due to its strong negative correlation with endoscopic severity (r = −0.754, *p* < 0.001), may serve as a serum non-invasive biomarker for monitoring disease activity in UC. Unlike CRP and fecal calprotectin, which show limited correlation with endoscopic findings, RvD1 may provide additional value by reflecting mucosal healing dynamics and the resolution phase of inflammation. The persistently low MaR1 levels observed even in remission suggest that it may serve as a potential biomarker for subclinical inflammation in UC. Persistent inflammation, despite clinical remission, is a critical factor in UC management. The significantly lower MaR1 levels in remission suggest that it may serve as an early indicator of unresolved mucosal inflammation, which could help guide treatment strategies. However, the specificity of MaR1 (60%) and RvD1 (80%) in distinguishing UC activity indicates that these biomarkers may not be sufficiently specific for standalone use. Instead, they may serve as complementary biomarkers alongside conventional inflammatory markers for more comprehensive UC monitoring.

In diseases with chronic inflammatory processes and metabolic disorders, pro-inflammatory cytokines are known to be elevated, while pro-resolving mediators are decreased [31]. Numerous human studies have investigated plasma MaR1 and RvD1 levels as biomarkers for assessing the activity of various diseases. When examining studies involving chronic inflammatory processes, Coras et al. investigated 41 patients with psoriatic arthritis, comparing disease activity scores with serum eicosanoids and RvD1 levels. They found that in patients with high disease activity, serum RvD1 levels were lower [32]. Our findings show that lower RvD1 levels are associated with higher disease severity, consistent with the results of this study. Karatay et al. found that patients with IBS had lower serum RvD1 levels and higher CRP levels than healthy controls. Both markers were identified as potential predictors for diagnosing IBS, particularly in patients with constipation-predominant IBS (IBS-C). Additionally, they showed that as RvD1 levels decreased, the severity of abdominal pain increased [21]. Özdemir et al. conducted a study involving 32 active RA patients, 33 in remission, and 20 healthy controls, measuring the blood levels of SPMs RvD1, RvE1, and LXA4, as well as inflammatory cytokines and chemokines. Similarly to our study, they reported that RvD1 levels, along with LXA4 and RvE1 levels, were notably reduced in active RA patients compared to those in remission and healthy controls, while inflammatory cytokine and chemokine levels were significantly elevated [19]. Wu et al. conducted a study involving 60 patients with postmenopausal osteoporosis (PMOP), a condition known to be associated with low-grade inflammation, as well as in 48 patients with osteopenia and 33 healthy individuals to evaluate serum MaR1 levels. The study found that serum MaR1 levels were lower in patients with PMOP or osteopenia than in healthy individuals, with the lowest levels observed in the PMOP group among the three groups. The researchers concluded that MaR1 could be utilized as a novel diagnostic marker for PMOP [33]. These findings support the hypothesis that MaR1 may serve as a biomarker for subclinical inflammation in chronic inflammatory conditions, as demonstrated in our study on UC. Song et al. conducted a study measuring serum RvD1 levels in patients with HT and healthy subjects. Logistic regression analysis showed a negative correlation between RvD1 and thyroid peroxidase antibodies (anti-TPO), which are linked to the severity of inflammation in HT patients. Additionally, the results demonstrated reduced serum RvD1 levels in HT patients, suggesting potential impairments in their inflammation resolution processes [17]. Fang et al. conducted a study that showed serum MaR1 levels were lower in patients with NAFLD compared to those without the disease and that elevated MaR1 levels were associated with a reduced risk of NAFLD. The study concluded that MaR1 levels could serve as a tool for predicting and preventing NAFLD [20]. Moreover, the persistently low MaR1 levels observed in clinically stable conditions such as NAFLD suggest the presence of ongoing inflammation even in the absence of overt clinical activity. In a study by Yavuz et al., 30 patients with polycystic ovary syndrome (PCOS), a condition linked to low-grade chronic inflammation, and 30 healthy controls were examined. The findings revealed that PCOS patients had elevated levels of malondialdehyde and CRP, alongside reduced MaR1 levels, than the control group. The study highlighted the promising role of MaR1 in being used as an early diagnostic biomarker for PCOS [34]. Tejera et al. conducted a study involving 26 patients with acute respiratory distress syndrome (ARDS); the serum levels of MaR1 and lipoxin A4, which play a role in lung injury resolution, were analyzed alongside lipid mediators involved in ARDS pathogenesis, such as thromboxane B2 and cysteinyl leukotrienes. The findings revealed that prolonged intensive care unit (ICU) stays and increased ventilator dependency were associated with low plasma levels of resolution-mediating factors. As a result, the study proposed that SPMs and other bioactive lipid mediators could be valuable biomarkers for the subphenotyping of ARDS [35]. Collectively, these findings underscore the universal role of SPMs in regulating inflammation across various diseases. Our study aligns with this body of research, demonstrating that MaR1 and RvD1 levels are significantly reduced in UC, particularly in active disease, and supporting their potential role as non-invasive biomarkers for mucosal inflammation. Given their ability to modulate neutrophil activity, cytokine production, and macrophage function, MaR1 and RvD1 could serve as valuable tools for monitoring disease activity and inflammation resolution in UC. Future research should assess whether these lipid mediators can be integrated into existing biomarker panels to improve non-invasive disease monitoring. Moreover, given the crucial role of SPMs in inflammation resolution, omega-3 DHA derivatives such as maresins and resolvins may represent promising therapeutic targets for modulating UC disease activity.

In this study, the classification of MES 1 as remission aligns with common clinical practice. However, it is important to consider that patients with MES 1 may still exhibit subclinical inflammation despite being classified as in remission. This is particularly relevant given our finding that MaR1 levels were significantly lower in the remission group compared to healthy controls, suggesting that residual inflammation may persist even when endoscopic healing appears to be achieved. This observation highlights the potential of MaR1 as a biomarker for detecting low-level inflammation that is not captured by endoscopic assessment alone. The role of MaR1 as a marker of subclinical inflammation has important implications for the long-term management of UC. Patients who are in clinical remission but exhibit low MaR1 levels may still be at risk of disease relapse, as unresolved inflammation could contribute to future disease flares. Recent perspectives on defining remission in UC have emphasized the importance of moving beyond endoscopic healing to histological and biological remission [36,37]. In this context, biomarkers such as MaR1 could play a crucial role in identifying patients who have achieved deeper levels of remission, potentially guiding more personalized treatment strategies. Future studies should explore the utility of MaR1 and other pro-resolving mediators in predicting long-term outcomes and tailoring therapeutic approaches in UC.

### Limitations

Despite the strengths of this study, including the evaluation of novel biomarkers in UC and the use of multiple inflammatory indices, certain limitations should be acknowledged. First, this study was conducted at a single center, which may limit the external validity of the results. Multicenter studies with a more diverse patient population could enhance the applicability of these findings. Second, there is an absence of fecal calprotectin measurements, which are widely used and validated biomarkers for assessing intestinal inflammation in UC. Although disease activity was assessed using serum inflammatory markers and endoscopic scores, fecal calprotectin could have offered additional validation of mucosal inflammation, particularly for differentiating active disease from remission. Third, due to the cross-sectional design, causal relationships between MaR1, RvD1, and disease activity cannot be fully established. Future longitudinal studies tracking changes in these lipid mediators across different disease states (active disease, remission, and relapse) would provide a deeper understanding of their role in UC pathogenesis. Additionally, emerging mediators such as the urotensin 2 receptor (UTS2R), which has been proposed as a predictor of steroid failure, could be explored in conjunction with MaR1 and RvD1 to assess their combined potential in monitoring disease progression and treatment response [38]. Fourth, although we performed a sub-analysis to assess the potential influence of 5-ASA therapy on MaR1 and RvD1 levels, the small number of non-5-ASA users in the remission group (*n* = 6) may limit the generalizability of these findings. Future studies with larger cohorts are needed to confirm our results. Lastly, the lack of tissue-level MaR1 and RvD1 measurements and the small sample size are key limitations of our study. Based on our a priori power analysis, we estimated that a minimum of 84 participants (28 per group) would be required to detect statistically significant differences between groups with an effect size of 0.40, a power of 80%, and a significance level of 0.05. However, our final sample size of 90 participants (30 per group) may still be underpowered, as the observed effect sizes for some biomarkers were smaller than anticipated. This could limit the generalizability of our findings and reduce the ability to detect subtle differences between groups. Future studies with larger cohorts are required to confirm our findings and enhance statistical robustness. Additionally, studies in which MaR1 and RvD1 levels are measured at the tissue level in endoscopic or colonoscopic biopsy specimens, combined with detailed analyses comparing these molecules to pro-inflammatory markers such as TNFα, IFNγ, IL17, and IL6, would provide significantly more reliable evidence regarding their potential as biomarkers in disease pathogenesis.

## 5. Conclusions

This study demonstrated that MaR1 and RvD1 levels were significantly reduced in UC patients, with the lowest levels observed in those with active disease. While RvD1 exhibited a strong negative correlation with disease severity, its specificity remains limited. Additionally, persistently lower MaR1 levels in the remission group compared to healthy controls may indicate residual inflammatory activity despite clinical stability. As DHA-derived lipid mediators, MaR1 and RvD1 play a crucial role in inflammation resolution, and their reduced levels may reflect an inadequate pro-resolving response, contributing to persistent inflammation. Since alterations in MaR1 and RvD1 levels are observed in various inflammatory conditions, their specificity for UC remains uncertain. However, their critical role in inflammation resolution suggests that they may serve as adjunct biomarkers for disease monitoring when used alongside conventional inflammatory markers. Further longitudinal studies are needed to explore whether MaR1 and RvD1 can contribute to comprehensive biomarker panels, complementing conventional inflammatory indices such as CRP and fecal calprotectin.

## Figures and Tables

**Table 1:** Patients’ clinical and demographic characteristics.

	Patients with Active UC X(*n* = 30)	Patients with UC in Remission (*n* = 30) ^b^	Control (*n* = 30) ^a^	*p*-Value
Age (years)	46.93 ± 17.87	47.03 ± 10.51	42.50 ± 10.85	0.337
Sex (male/female)	15/15	19/11	18/12	0.553
BMI (kg/m^2^)	29.79 ± 4.34	30.42 ± 3.70	30.04 ± 4.66	0.849
Smoking, *n* (%)	4 (13.3)	4 (13.3)	8 (26.7)	0.296
Duration of disease (months)	12 (0–63)	84 (36–120)	-	-
Medications (*n*)				0.073
None	12	6		
5-ASA	13	21		
5-ASA/Azatiopurine	5	3		
Mayo endoscopic subscore (*n*)				
MES 0	-	15	-	-
MES 1	-	15	-	-
MES 2	16	-	-	-
MES 3	14	-	-	-
ESR (mm/h)	18.50 (10–25.25) ^ab^	10.50 (8–16)	10 (8–11)	**<0.001**
Albumin (g/dL)	4.12 (3.96–4.72) ^b^	4.60 (4.33–4.77)	4.44 (4.23–4.54)	**0.043**
Leukocytes (×10^3^/μL)	7.01 (6.15–9.65)	6.45 (5.60–7.83)	6.52 (5.62–8.02)	0.099
CRP (mg/L)	12.19 (8.84–17.95) ^ab^	4.00 (1.86–8.38)	2.02 (1.15–4.02)	**<0.001**
Neutrophils (×10^3^/μL)	4.24 (3.05–6.62)	3.80 (3.04–4.76)	3.61 (3.18–4.65)	0.283
Lymphocyte (×10^3^/μL)	1.98 ± 0.62	2.18 ± 0.69	2.24 ± 0.71	0.297
Monocyte(×10^3^/μL)	0.51 ± 0.19	0.43 ± 0.15	0.45 ± 0.09	0.153
Platelets (×10^3^/μL)	330.50 (282.50–386.25) ^a^	295 (239.75–359.50)	280 (233.25–301.25)	**0.012**
HDL-C (mg/dL)	43.07 ± 7.26 ^ab^	48.37 ± 9.49	50.47 ± 10.09	**0.006**
NLR	2.55 (1.68–3.30) ^ab^	1.92 (1.42–2.22)	1.59 (1.26–2.15)	**0.009**
PLR	151.70 (127.28–241.68) ^a^	142.06 (109.58–172.60)	127.56 (88.41–156.34)	**0.019**
MHR	10.70 (7.87–15.89)	8.51 (6.44–12.06)	9.25 (7.88–11.26)	0.083
CLR	6.57 (3.60–12.44) ^ab^	1.99 (0.80–3.59)	0.94 (0.69–2.31)	**<0.001**
CAR	2.95 (2.00–3.99) ^ab^	0.83 (0.42–1.74)	0.46 (0.24–1)	**<0.001**
SII	946.33 (495.43–1175.75) ^ab^	585.51 (367.39–755.82)	424.86 (321.15–664.93)	**0.004**
SIRI	1.30 (0.69–1.71) ^ab^	0.77 (0.47–1.07)	0.72 (0.57–0.98)	**0.010**
MaR1 (pg/mL)	111.18 ± 20.86 ^ab^	125.38 ± 9.19 ^a^	141.20 ± 8.23	**<0.001**
RvD1 (pg/mL)	277.57 ± 32.04 ^ab^	351.24 ± 48.61	363.50 ± 60.61	**<0.001**

Data are presented as mean ± SD, median (25–75% inter-quartiles). Different superscript letters within a row indicate statistically significant differences between groups. Statistically significant values are marked in bold. UC, ulcerative colitis; BMI, body mass index; 5-ASA, 5-aminosalicylate; MES, mayo endoscopic subscore; ESR, erythrocyte sedimentation rate; CRP, C-reactive protein; HDL-C, high-density lipoprotein cholesterol; NLR, neutrophil to lymphocyte ratio; PLR, platelet to lymphocyte ratio; MHR, monocyte to high-density lipoprotein cholesterol ratio; CLR, C-reactive protein to lymphocyte ratio; CAR, C-reactive protein to albumin ratio; SII, systemic immune-inflammation index; SIRI, systemic inflammation response index; MaR1, maresin-1; RvD1, resolvin-D1. ^a^ Significantly different from the control group; ^b^ Significantly different from the remission group.

**Table 2:** Comparison of MaR1 and RvD1 levels between 5-ASA users and non-users in active and remission UC groups.

**Active**	**5-ASA Users (*n* = 18)**	**Non-Users (*n* = 12)**	***p*-Value**
MaR1 (pg/mL)	114.37 (97.22–132.44)	101.08 (91.14–113.99)	0.146
RvD1 (pg/mL)	294.19 (253.25–305.91)	283.33 (248.29–294.59)	0.267
**Remission**	**5-ASA Users (*n* = 24)**	**Non-Users (*n* = 6)**	***p*-Value**
MaR1 (pg/mL)	123.93 (118.93–132.08)	123.62 (117.21–132.38)	0.820
RvD1 (pg/mL)	359.14 (308.59–391.04)	332.62 (262.93–381.11)	0.273

Data are presented as mean ± SD, median (25–75% inter-quartiles). MaR1, maresin-1; RvD1, resolvin-D1.

**Table 3:** Accuracy of Mar1, RvD1, and other inflammatory markers in differentiating active UC from remission.

Parameter	AUC *	SE *	95% CI *	Cutoff	Sensitivity	Specificity	PPV	NPV	p **
NLR	0.672	0.060	0.566–0.801	>2.09	70.00%	73.3%	72.41%	70.96%	**0.012**
PLR	0.639	0.070	0.474–0.748	>194.52	43.30%	90.00%	80.00%	60.00%	0.065
MHR	0.675	0.043	0.557–0.726	>9.76	60.00%	63.3%	62.00%	61.29%	**0.043**
CRP	0.858	0.016	0.835–0.898	>6.04	93.30%	66.70%	73.68%	90.90%	**<0.001**
CLR	0.861	0.031	0.811–0.933	>3.46	83.30%	76.70%	80.64%	82.75%	**<0.001**
CAR	0.872	0.019	0.835–0.909	>2.03	76.70%	90.00%	88.46%	79.41%	**<0.001**
SII	0.700	0.064	0.597–0.848	>930.88	53.30%	90.00%	84.21%	65.85%	**0.009**
SIRI	0.708	0.067	0.585–0.848	>1.09	70.00%	80.00%	72.41%	71.87%	**0.007**
MaR1	0.717	0.077	0.576–0.879	<112.77	96.70%	60.00%	94.73%	70.73%	**0.003**
RvD1	0.906	0.040	0.827–0.984	<303.23	93.30%	80.00%	92.30%	82.35%	**<0.001**
Pairwise comparison ***		**Difference AUC**		**95% CI**		***p*-Value**			
CAR vs. CLR		0.012		−0.036–0.060		0.617			
CAR vs. CRP		0.014		−0.006–0.034		0.167			
CAR vs. RvD1		0.050		−0.178–0.078		0.444			
CLR vs. CRP		0.002		−0.050–0.054		0.949			
CLR vs. RvD1		0.062		−0.183–0.058		0.311			
CRP vs. RvD1		0.064		−0.195–0.067		0.339			

Statistically significant values are marked in bold. NLR, neutrophil-to-lymphocyte ratio; PLR, platelet-to-lymphocyte ratio; MHR, monocyte-to-high-density lipoprotein cholesterol ratio; CRP, C-reactive protein; CLR, C-reactive protein-to-lymphocyte ratio; CAR, C-reactive protein-to-albumin ratio; SII, systemic immune-inflammation index; SIRI, systemic inflammation response index; MaR1, maresin-1; RvD1, resolvin-D1. * AUC, standard error (SE), and confidence intervals (CI) were obtained using K-Fold Cross-Validation (K = 5) test sets. ** *p*-values were adjusted using the Benjamini-Hochberg method. *** DeLong’s test was used to compare the area under the ROC curves (AUCs).

**Table 4:** Accuracy of Mar1, RvD1, and other inflammatory markers in differentiating active UC from controls.

**Parameter**	AUC *	SE *	95% CI *	**Cutoff**	**Sensitivity**	**Specificity**	**PPV**	**NPV**	*p* **
NLR	0.661	0.060	0.544–0.778	>2.41	60.00%	86.70%	81.81%	68.42%	**0.008**
PLR	0.717	0.040	0.639–0.794	>144.46	60.00%	73.30%	69.23%	64.70%	**0.010**
MHR	0.694	0.034	0.628–0.761	>13.17	40.00%	93.30%	85.71%	60.86%	0.081
CRP	0.944	0.043	0.860–1.000	>6.23	93.30%	86.70%	87.50%	92.85%	**<0.001**
CLR	0.939	0.036	0.869–1.000	>3.42	83.30%	96.70%	96.15%	85.29%	**<0.001**
CAR	0.939	0.043	0.854–1.000	>1.28	93.30%	83.30%	84.84%	92.59%	**<0.001**
SII	0.694	0.060	0.576–0.812	>466.63	80.00%	66.70%	70.58%	76.92%	**0.003**
SIRI	0.717	0.027	0.664–0.769	>1.08	70.00%	86.70%	84.00%	74.28%	**0.014**
MaR1	0.889	0.066	0.759–1.000	<129.88	96.70%	80.00%	96.00%	82.85%	**<0.001**
RvD1	0.928	0.059	0.813–1.000	<303.35	96.70%	80.00%	96.00%	82.85%	**<0.001**
Pairwise comparison ***		**Difference AUC**		**95% CI**		***p*-Value**			
CRP vs. CLR		0.008		−0.014–0.029		0.480			
CRP vs. CAR		0.007		−0.008–0.021		0.360			
CRP vs. MaR1		0.069		−0.041–0.179		0.220			
CRP vs. RvD1		0.041		−0.045–0.128		0.351			
CLR vs. CAR		0.001		−0.029–0.026		0.937			
CLR vs. MaR1		0.061		−0.049–0.171		0.276			
CLR vs. RvD1		0.033		−0.056–0.123		0.466			
CAR vs. MaR1		0.062		−0.049–0.174		0.274			
CAR vs. RvD1		0.034		−0.053–0.122		0.439			
MaR1 vs. RvD1		0.028		−0.115–0.059		0.532			

Statistically significant values are marked in bold. NLR, neutrophil-to-lymphocyte ratio; PLR, platelet-to-lymphocyte ratio; MHR, monocyte-to-high-density lipoprotein cholesterol ratio; CRP, C-reactive protein; CLR, C-reactive protein-to-lymphocyte ratio; CAR, C-reactive protein-to-albumin ratio; SII, systemic immune-inflammation index; SIRI, systemic inflammation response index; MaR1, maresin-1; RvD1, resolvin-D1. * AUC, standard error (SE), and confidence intervals (CI) were obtained using K-Fold Cross-Validation (K = 5) test sets. ** *p*-values were adjusted using the Benjamini-Hochberg method. *** DeLong’s test was used to compare the area under the ROC curves (AUCs).

**Table 5:** Correlation between the MES and inflammatory indices in patients with active UC and UC in remission (*n* = 60).

Variable	Severity	
	r	p *
NLR	0.343	**0.009**
PLR	0.290	**0.028**
MHR	0.272	**0.036**
CRP	0.598	**<0.001**
CLR	0.614	**<0.001**
CAR	0.625	**<0.001**
SII	0.361	**0.007**
SIRI	0.357	**0.007**
MaR1	−0.407	**0.002**
RvD1	−0.754	**<0.001**

Statistically significant values are marked in bold. * *p*-values were adjusted using the Benjamani–Hochberg method; NLR, neutrophil-to-lymphocyte ratio; PLR, platelet-to-lymphocyte ratio; MHR, monocyte-to-high-density lipoprotein cholesterol ratio; CRP, C-reactive protein; CLR, C-reactive protein-to-lymphocyte ratio; CAR, C-reactive protein-to-albuminratio; SII, systemic immune-inflammation index; SIRI, systemic inflammation response index; MaR1, maresin-1; RvD1, resolvin-D1.

## Data Availability

Data available on request due to restrictions.
